# The rising roles of exosomes in the tumor microenvironment reprogramming and cancer immunotherapy

**DOI:** 10.1002/mco2.541

**Published:** 2024-04-07

**Authors:** Yu Wu, Wenyan Han, Hairong Dong, Xiaofeng Liu, Xiulan Su

**Affiliations:** ^1^ Clinical Medical Research Center of the Affiliated Hospital Inner Mongolia Medical University Hohhot China; ^2^ Clinical Laboratory the Second Affiliated Hospital of Inner Mongolia Medical University Hohhot China; ^3^ Clinical Laboratory Hohhot first hospital Hohhot China; ^4^ Hepatopancreatobiliary Surgery Department I Key Laboratory of Carcinogenesis and Translational Research (Ministry of Education/Beijing) Peking University Cancer Hospital and Institute Beijing China

**Keywords:** exosomes, immunotherapy, intercellular communications, tumor microenvironment, tumor therapy

## Abstract

Exosomes are indispensable for intercellular communications. Tumor microenvironment (TME) is the living environment of tumor cells, which is composed of various components, including immune cells. Based on TME, immunotherapy has been recently developed for eradicating cancer cells by reactivating antitumor effect of immune cells. The communications between tumor cells and TME are crucial for tumor development, metastasis, and drug resistance. Exosomes play an important role in mediating these communications and regulating the reprogramming of TME, which affects the sensitivity of immunotherapy. Therefore, it is imperative to investigate the role of exosomes in TME reprogramming and the impact of exosomes on immunotherapy. Here, we review the communication role of exosomes in regulating TME remodeling and the efficacy of immunotherapy, as well as summarize the underlying mechanisms. Furthermore, we also introduce the potential application of the artificially modified exosomes as the delivery systems of antitumor drugs. Further efforts in this field will provide new insights on the roles of exosomes in intercellular communications of TME and cancer progression, thus helping us to uncover effective strategies for cancer treatment.

## Graphical abstract

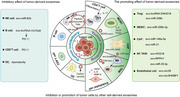

## 1 INTRODUCTION

The development of cancer is the result of various factors. In addition to gene mutations, tumor microenvironment (TME) also has crucial roles in cancer progression.^1^ TME is the circumstance that tumor cells reside, which consists of immune cells, endothelial cells, cancer‐associated fibroblasts (CAFs), peripheral blood vessels, extracellular matrix (ECM), as well as tumor cell itself.^2^ As vital components of TME, tumor‐infiltrating immune cells include adaptive immune cells (T cells and B cells) and innate immune cells (natural killer [NK] cells, dendritic cells [DCs], neutrophils, macrophages, and myeloid‐derived suppressor cells [MDSCs]).^3^ Cytokines from TME stimulate innate immune cells to regulate the recruitment, multiplication, and activation of lymphocytes, leading to tumor‐promoting or antitumor effects. Tumor cells has the ability to affect the components of TME, which is called as TME reprogramming. By reprogramming TME, tumor cells acquire immune escape.^4^ The mechanisms of tumor immune escape are complicated and become attractive topics in oncology. It is meaningful to study the communication between malignant cells and infiltrating cells in TME in order to develop new therapeutic methods.

Immunotherapy is a new type of strategies to treat cancers based on tumor immune escape mechanism. It is different from traditional treatment methods (surgery, chemotherapy, radiotherapy, and targeted therapy) which directly kill tumor cells. Immunotherapy aims to eliminate cancer cells by reactivate antitumor effect of the immune cells, with high specificity, safety, and effectiveness.^5^ Immune checkpoint inhibitors (ICIs) are currently the most studied immunotherapy. The important immune checkpoint molecules in tumor treatment are programmed cell death protein 1 (PD‐1) and cytotoxic T‐lymphocyte antigen 4 (CTLA‐4). The activation of immunosuppression aims to limit the autoimmune response when PD‐1 binds to its ligand programmed death ligand 1 (PD‐L1). However, with tumor progression, PD‐1/PD‐L1 binding reduces antitumor killing. CTLA‐4 is another inhibitory molecule besides PD‐1/PD‐L1. When CTLA‐4 interacts with CD80/CD86 of T cells, the inhibitory signals are transmitted to T cells in an effort to inhibit T cells‐related antitumor effect.^6^ The inhibitors of CTLA‐4 are studied in treating solid cancers. At present, a variety of monoclonal antibody drugs based on PD‐1/PD‐L1 and CTLA‐4 have been applied to treat cancers (Table 1). Recently, chimeric antigen receptor T cell (CAR‐T) therapy, as a kind of adoptive cell therapy, is applied to treat hematological malignancies. This therapy aims to genetically modify T cells and make the T cells directly target the specific antigens expressed in tumor cells. After activation, these modified T cells clear tumor cells with high efficiency. However, due to the lack of efficacy and safety of CAR‐T therapy, it is not used clinically to treat solid tumors. It is recently reported that the CAR‐T therapy recognizing claudin18.2 was used to treating gastric cancer (GC). The study entered clinical phase I trials and showed considerable efficacy.^7^ Cancer vaccines is another strategy by using tumor‐related antigens to improve the antitumor activity of T cells and establish sustained antitumor memories. At present, this method is under research and is not used in the therapeutics of solid tumors.^8^

**TABLE 1:** Approved ICIs for cancer treatment until 2023.

Drug	Name	Tumor	Target	References
Nivolumab	Opdivo	NSCLC, GC, EADC, CRC	PD‐1	^16^
Pembrolizumab	Keytruda	GC, CRC, LC	PD‐1	^16^
Sintilimab	Daboshu	NSCLC, HCC	PD‐L	^17^
Atezolizumab	Tecentriq	BCa, HCC	PD‐L1	^17^
Imfinzi	Durvalumab	SCLC	PD‐L1	^17^
Ipilimumab	Yervoy	melanoma	CTLA‐4	^18^
Imjudo	Tremelimumab	HCC	CTLA‐4	^17^

Abbreviations: BCa, bladder cancer; CRC, colorectal cancer; EADC, esophageal adenocarcinoma; GC, gastric cancer; HCC, hepatocellular carcinoma; ICIs, immune checkpoint inhibitors; LC, lung cancer; NSCLC, non‐small cell lung cancer; SCLC, small cell carcinoma of lung.

**FIGURE 1:**
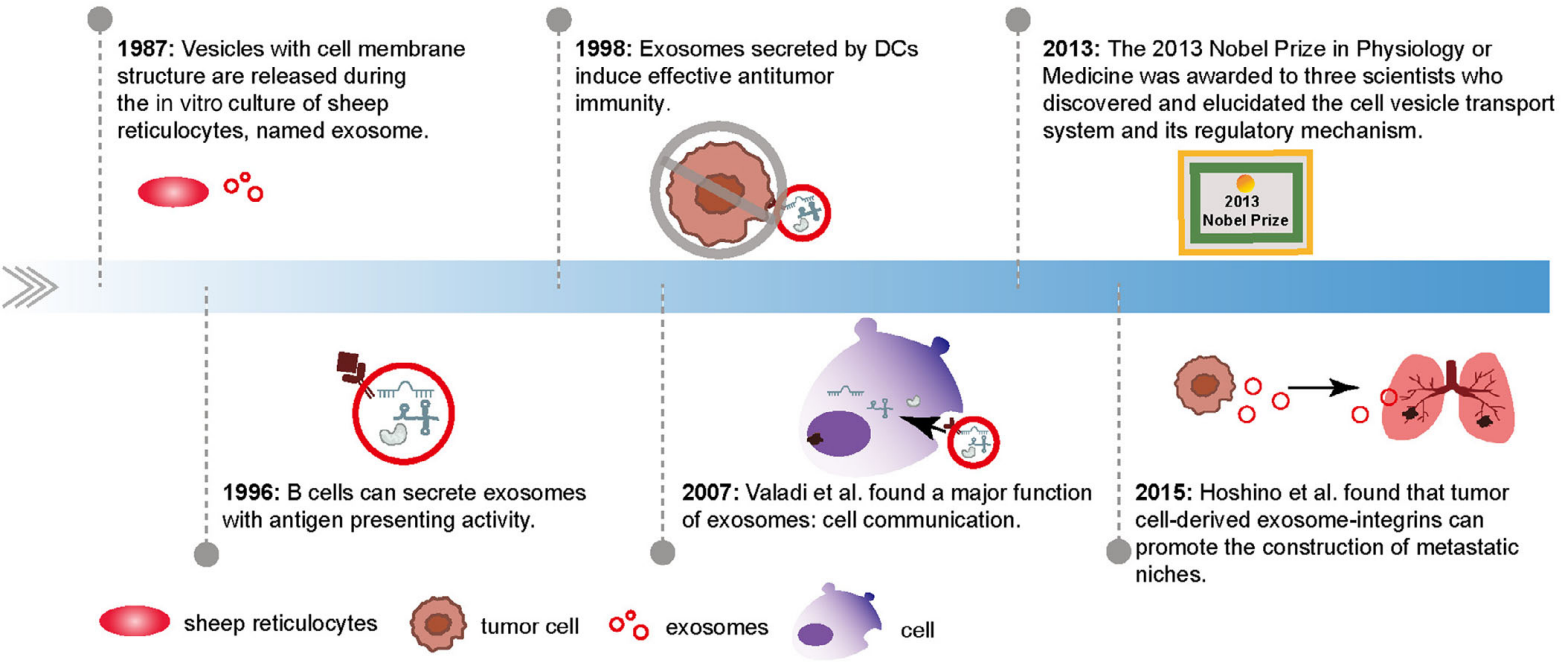
The timeline of major events in the history of exosome research. Researchers isolated a vesicle with cell membrane structure from the supernatant of sheep reticulocyte and named it exosome.^231^ Initially, it was thought that exosomes were simply waste products generated during cellular metabolism. With the improvements in exosome isolation techniques and identification methods, scientists found that immune cells including B cells,^232^ DCs also secrete exosomes.^233^ These exosomes secreted by B cells and DCs encapsulate major histocompatibility complex (MHC) class II molecules and T cell costimulatory molecules to exert antigen presentation activity.^233^ Subsequently, it is reported that exosomes‐carried non‐coding RNAs (ncRNAs) are required for intercellular communications.^234^ These data indicate that exosomes play pivotal roles in cell–cell communication. In 2013, two scientists who discovered and described the cellular vesicle transport system and its regulatory mechanisms won the Nobel Prize in Physiology or Medicine. This further led to an explosion of research on exosomes. In 2015, it is reported that the resident cells in tumor metastatic target organs were able to take up tumor cell‐derived exosomal integrins to promote the construction of metastatic niche.^235^ Subsequently, more and more studies confirm the regulatory value of exosomes in TME.

Exosome plays vital roles in the communications between the infiltrating cells and cancers cells in TME. Usually, exosomes refer to the nanoscale vesicles released from a variety of cells and stably exist in many kinds of body fluids, with a lipid bilayer structure. The components of exosomes mainly include proteins, nucleic acids, and lipids.^9^ Lipid is an important component of exosome membrane structure. Proteins in the surface of exosome, such as Annexin, contribute to membrane fusion. Currently, heat shock proteins (HSP60, and HSP70) and tetrapeptides (CD63, CD81, and CD9) are identified as protein markers of exosomes.^10^ Nucleic acid is the main “cargo” of exosomes, including DNA and RNA.^11^ The discovery of exosomes can be traced back to the 1980s (Figure 1). As a messenger for the cell–cell communication, exosome has important impacts on tumor progression by transporting various signals.^12^ Cancer cells regulate immune cells by releasing exosomes into the extracellular space for reprogramming microenvironment, while exosomes originated from stromal cells also assist cancer cells into invasive phenotypes.^13^ More and more studies have found that exosomes possess unique advantages as drug carriers due to their low toxicity and low immunogenicity.^14^ Exosomes can be engineered to enhance their drug load and targeting capabilities, making them as a promising therapeutic option. It is important to further discuss their potential benefits.^15^

Here, we review recent advances in the exploration of the mechanisms by which exosomes participate in communication between cancer cells and various stromal cells and discussed possible targets that could be used to improve the efficacy of immunotherapy. Additionally, we also discussed the potential role of engineering exosomes in cancer treatment. The aim is to provide new insights into the basic research and treatment of exosomes in cancer.

## 2 EXOSOMES REGULATE THE COMMUNICATIONS BETWEEN TME AND CANCER CELLS

**FIGURE 2:**
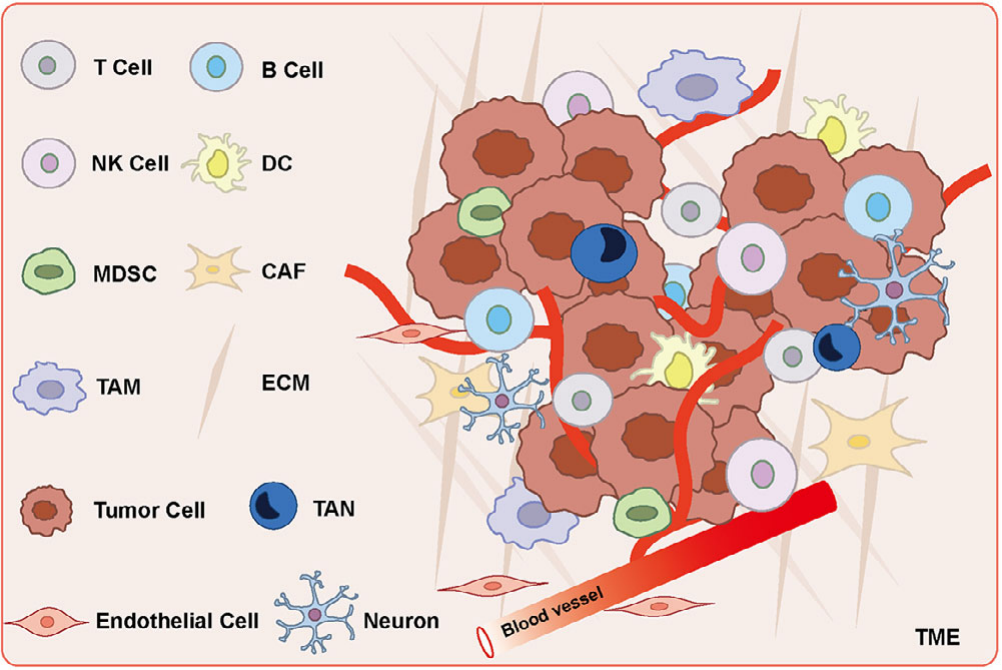
Composition of tumor microenvironment. TME is the circumstance that tumor cells reside, which consists of immune cells, endothelial cells, CAFs, peripheral blood vessels, ECM, as well as tumor cell itself. NK, natural killer; DC, dendritic cell; MDSC, myeloid‐derived suppressor cell; CAF, cancer‐associated fibroblast; TAM, tumor‐associated macrophage; TAN, tumor‐associated neutrophils; ECM, extracellular matrix.

The composition of TME is shown in Figure 2. The cells in TME intertwine to form a huge interaction network to affect the occurrence and progression of tumors. In order to analyze the pathogenesis of tumors and develop new therapeutic drugs, it is necessary to understand how TME interacts with tumor cells. As a media for cell‐to‐cell communication, exosomes are undoubtedly at the forefront. Exosomes transform TME into a tumor‐promoting state by transmitting signals, thereby forming a microenvironment conducive to tumor cell growth or a premetastatic niche for cancer cell colonization. Therefore, the exploration of communication effect of exosomes between TME and tumor cells is helpful to find new immunotherapy targets.

### 2.1 Exosomes regulate the communication between T cells and cancer cells

T cells, as important members of lymphocytes, have the ability to kill the indicated cells or assist B cells to produce antibodies. Tumor cells use exosomes to promote T cell dysfunction since T cells are the main force of antitumor effect. The discoveries of the related mechanisms will aid in developing new treatments to restore T cell function.

CD8^+^ T cells function in killing intercellular infections or malignant cells, as well as helping to eliminate pathogens through secreting cytokines or directly clearing infected cells.^19^ Infiltrating CD8^+^ T cells in TME are positively correlated with the outcome of patients, but in lots of cases, CD8^+^ T cells are exhausted.^20^ The strategy to upregulate the ratio of CD8^+^ T cells in tumors and enhance their activity is a promising method for treating tumors.^21^ Exosomes have been found to promote the depletion or dysfunction of CD8^+^ T cells in various tumors, including hepatocellular carcinoma (HCC),^22^ bladder cancer (BCa),^23^ and papillary thyroid carcinomas.^24^ It is observed that tumor cells use exosomes to regulate the cytokine secretion or gene expression of infiltrating CD8^+^ T cells in order to repress their effects. For example, HCC cell‐derived exosome 14‐3‐3 protein zeta (14‐3‐3ζ) promotes the exhaustion phenotype of CD8^+^ T cells, and upregulates the differentiation of naive T cells into Treg.^25^ CD8^+^ T cells express immunosuppressive gene *FOXP3* and immunosuppressive factor IL‐10 after cultured with cancer cell‐derived exosomes.^26^ When CD8^+^ T cells ingest exosomal circCCAR1^22^ released from HCC cells, exosomal circUSP7^27^ secreted by non‐small cell lung cancer (NSCLC) or O‐GlcNAc transferase^28^ derived from esophageal cancer (EC) stem cells, the degradation of PD‐1 is inhibited, which further represses the effects of CD8^+^ T cells. In tumor cells, HRS phosphorylation promotes the secretion of immunosuppressive exosome and inhibits the infiltration of CD8^+^ T cells into tumors.^29^ The engineered peptide targeting this feature can destroy the envelope of tumor‐derived exosomes, reducing the level of the circulating exosomes and restoring the function of CD8^+^ T cells.^30^ This suggests that targeting tumor‐derived exosomes with drugs enhance the antitumor effects of immunotherapy in a synergistic manner.

CD4^+^ T cells include helper T cell and regulatory T cells (Treg). The former usually play an auxiliary effect in antitumor response. The infiltrating T helper cells in the TME are highly heterogeneous, including multiple subsets (Th1, Th2, Th17). T helper type 1 (Th1) expresses a variety of cellular factors (IL‐2, IFN‐γ, and TNF) and plays a critical role in antineoplastic response.^31^ T helper type 2 (Th2) secretes IL‐5 to activate eosinophils to fight against extracellular pathogens. In order to maintain homeostasis, Th2 antagonizes the function of Th1.^32^ Previous studies have suggested that T helper type 17 (Th17) has an indirect antitumor effect.^33^ However, Th17 induce impaired immune function, and IL‐17 secreted by Th17 is participated in tumorigenesis and metastasis.^34^ The increased infiltration of Th17 cells in TME is tightly correlated with the poor outcome of patients.^35^ At present, the research on exosomes secreted by tumor cells is focused on inducing Th17 differentiation. For example, exosomal miR‐451 secreted by GC cells under low glucose conditions increase the proportion Th17 cells.^36^ Exosomes secreted by colorectal cancer (CRC) cells deliver lncRNA CRNDE‐h to CD4^+^ T cells to upregulate the excretion of IL‐17 to induce the differentiation from naive CD4^+^ T cells into Th17 cells.^37^ Targeted inhibition of Th17 differentiation and IL‐17 secretion helps to achieve antitumor therapeutic effects.^38^

Tregs are well‐known immunosuppressive cells that have important effect in tumor immune evasion. The infiltration of Treg facilitates the cell proliferation and drug resistance of cancer cell.^39^ Tumor‐derived exosomes often carry signals to promote Treg proliferation, which may be a target for the discovery of new immunotherapy.^40^ Tumor‐derived exosomes in lung cancer create a premetastatic niche of immune tolerance by activating Tregs.^41^ Exosomal miR‐208b from CRC cells supports the proliferation of Tregs and induces oxaliplatin resistance.^42^ Breast cancer (BC)‐derived exosomes transmit lncRNA SNHG16 to induce CD73^+^ γδ1 Treg cells expansion.^43^ In conclusion, targeting the interaction between tumor exosomes and Tregs could be a new approach to improving immunotherapy.

In addition to the above common T cell classification, it can also be divided into αβ T cells (such as CD4, CD8) and gamma delta (γδ) T cells according to the difference of T cell receptor (TCR). Vγ9Vδ2 T cell is a main subset of γδT cells with strong antitumor activity. GC‐secreted exo‐miR‐135b‐5p reduces the viability of Vγ9Vδ2 T cells by regulating specific protein 1 (SP1) and reduces the excretion of IFN‐γ and TNF‐α. Targeting the exosomal miR‐135b‐5p/SP1 axis is probably used in increasing the efficiency of Vγ9Vδ2 T cells‐related immunotherapy in GC.^44^

Various classifications assign different roles to T cells in tumor treatment. The inhibition of tumor‐secreted exosomes can reactivate antitumor CD8^+^ T cells and inhibit tumor‐promoting Th17 and Treg cells. This is significant for improving immunotherapy. Furthermore, the engineering exosomes constructed using exosomes secreted by CAR‐T cells present significant efficacy in treating lung cancer.^45^ This suggests that the combination of immunotherapy and engineering exosomes in the treatment of cancer is attractive.

### 2.2 Exosomes regulate the communication between B cells and cancer cells

The roles of B cells in the antitumor effect have recently received attention. B cells and plasmacyte fight against cancer cells through antibody‐dependent cytotoxicity (ADCC) and alexin cascade activation.^46^ Additionally, B cells deliver antigen to stimulate T cells and localize to the tertiary lymphoid structure to induce particular T cell action.^47^ It has been found that B cells increase the efficacy of immunotherapy, especially for ICI‐resistant human cancers.^48^ In clinical cohort studies, B cells from ICI responders were in clonal expansion and unique functional status,^49^ and patients in the B cell‐rich immune subgroup were more sensitive to anti‐PD‐1 treatment.^50^ However, tumor‐derived exosomes upregulate the level of PD‐1 and LAG3 in B cells and counteract the activity of B cells.^51^ CRC‐derived exosome lncRNA HOTAIR downregulates the functions of CD8^+^ T cells by inducing the regulatory characteristic polarization of B cells.^52^ Regulatory B cell is a subgroup of B cells with immunosuppressive function, which promotes immune escape through repressing the activity of T cells and NK cells,^53^ as well as regulating the differentiation of Tregs.^54^ Tumor‐derived exosomal HMGB1 promotes liver cancer immune escape by promoting the multiplication of TIM‐1^+^ regulatory B cells.^55^

In recent years, the role of B cells in antitumor response has been discovered. B cells act as allies of T cells to coordinate the immune response. Tumor exosomes tend to inhibit the activity of antitumor B cells and activate tumor‐promoting regulatory B cells. However, the specific mechanism of action is not yet clear, and further study is needed.

### 2.3 Exosomes regulate the communication between NK cells and cancer cells

NK cells, as the name suggests, kill target cells autonomously. The activation receptors of NK cells, such as NCR1, NCR2, NKG2D, NKG2C, CD244, Ly49D, and Ly49H, mediate the function of NK cells by recognizing pathogen‐derived ligands.^56^ NK cells participate in the defense against pathogens and tumors by releasing perforin and granzyme family proteins.^57^

Exosomes produced by NK cells (NK‐exo) also induce tumor cell death.^58^ DNAX Accessory Molecule‐1 (DNAM1) receptor is critical in NK cell‐related tumor cell recognition and killing.^59^ Study has found that the receptor also exists on the surface of NK‐exo, which recognizes ligands on target cells, facilitates internalization of exosomes, and participates in NK‐exo mediated cytotoxicity.^60^ In addition, these exosomes also express the proteins involved in killing, such as granzyme B, perforin, TRAIL, and FasL.^58^ NK‐exo shows active treatment effects in orthotopic and subcutaneous liver cancer mice.^61^

Although NK cells have strong cytotoxicity to kill cancer cells, the infiltrating NK cells in solid tumors are scarce.^62^ More and more studies reveal that immunotherapy based on NK cells has a good antitumor effect.^63^, ^64^, ^65^ The NK cells derived from pluripotent stem cells enhance the expression of cytokines and produce strong cytotoxicity to tumors, cooperated with anti‐PD‐1 antibodies and T cells.^66^ CAR‐NK cell therapy displays the good clinical results in hematological malignancies.^67^, ^68^ After receiving anti‐CD19 decorated CRA‐NK cell therapy, 73% of patients with recurrent or intractable CD19‐positive malignancy (chronic lymphocytic leukemia or non‐Hodgkin's lymphoma) benefited from the therapy.^69^ However, due to the tumor immune escape caused by TME, the efficacy of NK cell‐based therapy remains limited in most clinical trials in solid tumors.^57^ Tumor cells frequently induce the exhaustion of the infiltrating NK cells by releasing various immune escape molecules, including exosomes.^40^ For example, exosomes secreted by cancer cells upregulate INHBC to protect CRC cells from NK cell killing.^70^ Exosomes secreted by poorly differentiated GC cells are internalized by NK cells in the lungs to promote the immune microenvironment to form a metastatic niche to promote tumor metastasis.^26^ Exosomal circuxHRF1^71^ and miR‐92b^72^ secreted by HCC cells cause NK cell dysfunction and promote resistance to anti‐PD1 treatment.

Although tumor‐derived exosomes inhibit NK cells, engineering NK‐exo reverse this immunosuppression by loading drugs. When used as a carrier to deliver cisplatin, NK‐exo enhances the cytotoxicity of chemotherapeutic drugs to drug‐resistant ovarian cancer (OC) cells.^73^ This suggests that we should pay attention to the potential of NK‐exo in drug loading. Recent studies have also constructed CAR‐NK engineering exosomes for the treatment of breast carcinoma (BC),^74^ once again reminding us of the research trend of engineering exosomes.

### 2.4 Exosomes regulate the communication between DCs and cancer cells

DCs have strong antigen presenting function, which is the strongest among the known cells.^75^ After activation, DCs use surface receptors (such as phagocytosis receptors, and endocytosis receptors) to ingest antigens, and display antigens to T cells.^76^ DCs in TME are indispensable for effector T cells transport and adoptive cell transfer therapy. The lack of CD103^+^ DCs in TME will restrain the tumor‐killing effect of T cells, resulting in immune evasion.^77^

DCs‐derived exosomes retain the property of parental cells to expand the proportion of CD8^+^ T cells, and activate T cells, which are beneficial to ameliorate the antineoplastic response of TME in orthotopic cancer mice.^78^ In addition, exosomes secreted by DCs infected with *Toxoplasma gondii* repress the growth of tumors in mice by decreasing the infiltration of MDSCs.^79^ Exosomes secreted by Epstein–Barr Virus‐associated GC (EBVAGC) cells attenuate the maturation of DCs and the decrease of the amount of CD83‐positive mature DCs is related to poor prognosis in EBVAGC patients.^80^

Antitumor vaccines based on DCs have great potential in tumor therapy and are being developed for cancer immunotherapy.^81^ DC‐exosomes carry major histocompatibility complex (MHC) surface molecules and peptide‐loaded major histocompatibility complex (pMHC), which function in presenting antigen and inducing antigen‐specific T cell responses.^82^ In HCC, compared with microwave ablation (MWA) alone, MWA combined with DC‐exosomes largely improved the treatment outcome in mice model.^83^ The treatment with DC‐exosomes rich in α‐fetoprotein upregulated the infiltrating CD8^+^ T cells in TME, induced antigenic specificity immune reaction, and significantly improved the survival periods of HCC mice.^78^ Therefore, it is possible that DC‐exosomes are expected to completely or partially mimic mature DCs and become natural antitumor adjuvants or tumor vaccines.

The natural advantages of DCs antigen presentation make it as a tumor vaccine for antitumor therapy.^84^ Since this vaccine is noninvasive with low side effects, it is attractive in the research of tumor vaccines.^85^ A vaccine expressing Retinoic acid early inducible‐1γ (RAE‐1γ) is prepared for chronic myeloid leukemia (CML), called CML‐RAE‐1γ‐Dex. In vitro studies showed that the vaccine significantly improves anti‐CML efficacy by promoting the proliferation and effector function of NK cells and CD8^+^ T cells.^86^ Although many studies have confirmed the effectiveness of this method,^87^, ^88^, ^89^ the difficulty of collecting natural DCs limit its development because of the extremely low number in tumor.^90^ DC‐exosomes inherit the antigen‐presenting activity of parental cells and have strong antitumor activity, which may be an alternative to DCs vaccine.^81^

### 2.5 Exosomes regulate the communication between MDSCs and cancer cells

MDSCs are subsets of immune cells originated from neutrophils or monocytes in the pathological state and have strong immunosuppressive ability.^91^ In the TME, MDSCs communicate with other immune cells to repress the function of T cells and NK cells, promote the expansion of Tregs and support tumor progression.^92^ The proportion of MDSCs are correlated with the adverse prognosis of cancer patients.^93^ Usually, MDSCs can be roughly assigned to two subsets: mononuclear MDSCs (M‐MDSCs) and polymorphonuclear MDSCs (PMN‐MDSCs).^94^ They promote tumor cells to become resistant to immunotherapy by inhibiting the antitumor activity, which is a major disturbance to cancer immunotherapy.^95^ Targeted inhibition of M‐MDSCs development significantly improve the efficacy of ICI therapy and prolong the survival time of tumor‐bearing mice.^96^

The communication between MDSCs and tumor cells is the driving force for the deterioration of various tumors.^97^, ^98^ Exosomes play a role in transmitting cancer‐promoting signals in this process.^99^, ^100^ Exosomes produced by MDSCs (MDSCs‐exos) acquire the analogous function to parental MDSCs in modulating tumor immunity.^93^ G‐MDSCs‐exos that are rich in miRNA‐143‐3p promote the proliferation of lung cancer cells by targeting ITM2B.^101^ Furthermore, the S100A9 present in MDSCs‐exos not only enhances the stemness of CRC cells^102^ but also contributes to the advancement of castration‐resistant prostate cancer.^100^ Moreover, exosomes originated from tumor cells induce the accumulation of MDSCs. When exosomes are internalized by MDSCs, they promote the secretion of cytokines (IL‐6, vascular endothelial growth factor [VEGF], prostaglandin E2).^103^ Exosomes secreted by various tumors, including renal cancer,^97^ BC,^98^ and glioma,^104^ have been shown to activate the immunosuppressive function of MDSCs. Among them, BC‐derived exosomal miRNA‐200b‐3p regulates the expression of C‐C motif chemokine ligand 2 (CCL2) in lung epithelial cells, promoting specific organ metastasis.^105^ Furthermore, tumor‐associated exosomes carry macrophage migration inhibitory factor, which activates the differentiation of MDSCs and promotes the progression of pancreatic cancer.^106^ There are also interactions between other stromal cells in TME and MDSCs. For example, esophageal squamous cell carcinoma (ESCC)‐associated fibroblasts activate signal transducing activator of transcription 3 (STAT3) signaling by paracrine exosomal miR‐21 to induce monocytes to transform into M‐MDSCs, thereby promoting cisplatin resistance.^107^

The characteristics of MDSCs participating in tumor immune resistance in TME make it an important target for the treatment of tumors.^108^ Exosomes from tumor cells are conducive to the reactivation and proliferation of MDSCs. Exosomes secreted by MDSCs support tumor development by repressing the function of T lymphocytes, thus forming a vicious circle. Inhibition of exosome secretion may help overcome tumor progression.^93^ However, the current research on exosomes modulating the communication between tumor cells and MDSCs is limited, and further studies are helpful to develop effective targets for tumor immunotherapy.

### 2.6 Exosomes regulate the communication between tumor‐associated macrophages and cancer cells

Tumor‐associated macrophages (TAMs) are simply defined as macrophages infiltrating in TME, which are the immune cells most likely to be “repulsed” by tumor cells.^109^ For tumor immunity, macrophages are divided into “good” and “bad” macrophages. The former are M1‐type macrophages that exert antitumor activity and kill cancer cells by directly mediating cytotoxicity or ADCC.^110^ The “bad” macrophages are defined as the M2‐type that facilitate tumor progression by repressing T cell‐mediated antitumor response.^110^ Since TAMs have strong plasticity, the strategy reprogramming TAMs into M1‐type is helpful for improving CD8^+^ T cell activity and delaying tumor growth.^111^ M2‐type TAMs are correlated with poor prognosis of cancer patients and immunotherapy resistance,^112^ which is one of the causes explaining the poor outcome of ICIs in solid tumors. Targeting the recruitment, activation, and polarization of TAMs in combination with immunotherapy improve the efficacy of ICIs.^113^ A deeper understanding of the mechanism of TAM's mutual regulation with tumor cells provide new insights into improving immunotherapy.

Tumor cells induce the M1 macrophages to polarize to the M2‐type by releasing various factors, thereby inducing immune escape of cancer cells.^114^ Exosomes are one of important signals released by tumor cells in this progression. miRNAs are packaged into exosomes of cancer cells, and then transported to TAMs to promote M2 polarization.^115^ For example, the exosome‐related miR‐25‐3p,^116^ miR‐130b‐3p,^116^ miR‐4255p,^116^ miR‐934,^115^ and miR‐301a‐3p^117^ downregulate *PTEN* expression level and enhance PI3K/Akt to motivate macrophage differentiation into M2‐type in tumor. Recent papers have reported that long noncoding RNAs (lncRNAs) also have a certain role in promoting macrophage M2 polarization, such as HCG18^118^ and RPPH1.^119^ In addition, exosomes transport PD‐L1 to TAMs in order to inhibit CD8^+^ T cells in HCC.^120^

M2 macrophages also use exosomes to promote tumor progression, thus forming a vicious cycle. M2 macrophage‐derived exosomes (MDEs) reduced the expression of tumor suppressor gene *BRG1* or *TIA1* by miRNAs to facilitate tumor progression.^121^, ^122^ MDEs have been studied in various tumors, promoting not only tumor angiogenesis in pancreatic ductal adenocarcinoma^123^ but also the progression of lung adenocarcinoma by delivering miR‐942.^124^ Furthermore, exosomes transfer functional apolipoprotein E to GC cells, motivating the PI3K/Akt signaling and enhancing GC cell migration.^125^ Contrary to the effects of MDEs, exosomes derived from M1 TAMs inhibit tumor progression. For instance, exosomes secreted by M1 TAMs that are rich in tumor suppressor miR‐628‐5P inhibit the progression of HCC by suppressing the m^6^A modification of circFUT8.^126^

Reprogramming TAMs into M1‐type to enhance antitumor activity is a potential method to improve immunotherapy, given the heterogeneity of TAMs. Three potential methods for exosome‐based TAMs reprogramming exist. The first is to inhibit the secretion of tumor‐associated exosomes to reduce TAMs M2 polarization. The second is to inhibit MDEs to prevent tumor progression. The third is to use exosomes secreted by M1‐type TAMs to design drugs that promote M1 polarization.

### 2.7 Exosomes regulate the communication between tumor‐associated neutrophils and cancer cells

Tumor‐associated neutrophils (TANs) in TME are characterized by heterogeneous phenotypes and functional diversity.^127^ TANs have two phenotypes. In the early carcinoma, they are chiefly proinflammatory N1‐type, which is related to innate immune response.^128^ They are characterized by high levels of reactive oxygen species and increased activation of NF‐κB signaling.^129^ With the continuous development of tumors, anti‐inflammatory N2‐type increases the secretion of matrix metalloproteinase 8 or 9 (MMP8, MMP9), IL‐1β and IL‐6 to promote tumor invasiveness.^130^

N2‐type TANs are usually associated with poor outcomes of patients, mainly in promoting tumor growth, angiogenesis and metastasis.^131^ The roles of TANs on tumor cells are mediated through interacting with CD8^+^ T cells. TANs promote immunosuppression by accelerating apoptosis of CD8^+^ T cells.^132^ In addition, exosomes from cancer cells enhance the tumor‐promoting effect of TANs. N2‐type TANs recruited in premetastatic niche promote brain metastasis of lung cancer by secreting the exosomeal miR‐4466.^133^ The exosome piRNA‐17560 derived from senescent neutrophils promotes chemoresistance and epithelial–mesenchymal transition in BC.^134^ Neutrophils have an important role in cancer‐related inflammation, which not only upregulate immune response but also result in immunosuppression.^135^ Neutrophil extracellular traps (NETs) are a kind of network configuration composed of histones, DNA, and antibacterial proteins secreted by activated neutrophils after certain stimulation, which are responsible for capturing and killing extracellular pathogens.^136^ NETs trigger tumor inflammatory response and promote tumor development.^137^ Excessive NETs in HCC tissues are internalized into HCC cells and promote TLR4/9‐COX2 signaling to cause inflammatory response and induce metastasis of HCC cells.^138^ The increase of NETs formation in tumors can be caused by exosomes secreted by tumor cells.^139^ The transfer of mutant *KRAS* protein from CRC cells to TANs promotes the secretion of IL‐8, induces the infiltration of TANs and the formation of NETs, and finally enhances the deterioration of CRC.^139^

At present, the role of exosomes in the communication between TANs and tumor cells has not attracted enough attention. However, the potential therapeutic effect of TANs‐derived exosomes is hard to ignore. Researchers used superparamagnetic iron oxide nanoparticles to modify exosomes secreted by neutrophils to construct engineering exosomes and induce apoptosis of tumor cells by delivering cytotoxic proteins and activating the caspase signaling pathway.^140^ This suggests that exosomes secreted by neutrophils have the potential for engineering.

### 2.8 Exosomes regulate the communication between CAFs and cancer cells

CAFs are an important type of stromal cells in the TME and have a supporting role in cancer development. CAFs are mainly differentiated from mesenchymal stem cells (MSCs), pericytes or fibroblasts.^141^ As a bridge between cells, exosomes participate in the transformation of various cells into CAFs in tumors. TGF‐β from exosomes secreted by cancer cells interacts with receptors on human umbilical cord MSCs to activate the Smad signaling and induce MSCs to differentiate into CAFs.^142^ Bone morphogenetic proteins^143^ and miR‐21^144^ in exosomes induced the transformation of pericytes and hepatocyte stellate cells into CAFs, respectively. CAFs reshape the state of TME by crosstalk with cancer cells and other components, thereby contributing to the growth, metastasis and immune evasion of cancer treatment.^145^ CAFs recruit suppressive cells (such as MDSCs) by secreting chemokines and cytokines, containing TGF‐β, IL‐6, C‐X‐C chemokine ligand 12 (CXCL12), and prostaglandin E2.^146^

Besides, exosomes also participate in the communication of CAFs with other cells.^147^ Tumor‐secreted exosomes support the proliferation of cancer cells by reprogramming fibroblasts.^148^ For example, exosomal miR‐146a‐5p and miR‐155‐5p^149^ secreted by CRC cells promote tumor growth after activating CAFs. miR‐1247‐3p released by HCC cells induces CAFs activation and promotes lung metastasis of HCC.^150^ Exosomal miR‐141, secreted by OC cells, reprograms stromal fibroblasts into proinflammatory CAFs, promoting metastasis and colonization.^151^ Multiomics studies have found that the reason why CAFs‐derived exosomes promote tumor development is that they are rich in THBS2, a key factor that promotes the invasiveness of early lung adenocarcinoma.^152^

The communication between cancer cells and CAFs is mutual, and the exosomes released from CAFs carry a large number of tumor‐promoting signals. Low expression of tumor suppressor factors such as miR‐34,^153^ miR‐139,^154^ in exosomes secreted by CAFs inhibit the development of tumor. High expression of cancer‐promoting factors, such as miR‐3656^155^ and miR‐92a‐3p,^156^ promote tumor progression. CAFs‐derived miR‐500a‐5p promotes BC cell proliferation by targeting USP28.^157^ LncRNA NEAT1 derived from CAFs promotes the progression of endometrial cancer through miR‐26a/b‐5p‐mediated STAT3/YKL‐40 pathway.^158^ CAFs‐derived exo‐circEIF3K is secreted under hypoxia stimulation and promotes CRC progression.^159^ CAFs‐derived exosome miR‐24‐3P induces methotrexate resistance by inhibiting the CDX2/HEPH axis.^160^ Exo‐lncRNA CCAL binds to the mRNA‐stabilizing protein HUR to induce oxaliplatin and 5‐fluorouracil resistance in CRC cells.^161^

The important regulatory role of CAFs in TME makes it a new target for anticancer immunotherapy. Targeting microfibril‐associated protein 5 (MFAP5) in CAFs helps to ameliorate the susceptibility of cancer cells to PD‐L1‐based immune‐chemotherapy.^162^ In addition, targeting CAFs also upregulates the sensitivity to CAR‐T cell therapy.^163^ It is valuable to explore the related mechanism of CAFs remodeling on TME for the development of immunotherapy targets. As a communication tool, exosomes not only carry the activation signals released by tumor cells to stimulate the differentiation of CAFs, but also provide the tumor‐promoting signals secreted by CAFs to cancer cells to promote tumor immune escape. Although CAFs can also construct tumor‐promoting TME by modulating other immune cells, the function of exosomes in this process is still unclear.^147^ This is a promising research direction for the subsequent study of new immunotherapy targets.

### 2.9 Exosomes regulate the communication between endothelial cells and cancer cells

Angiogenesis and vascular permeability are crucial in promoting tumor growth and metastasis. Endothelial cells are the main cells driving angiogenesis, which migrate and proliferate against blood flow to form new blood vessels. The channels provided by neovascularization promote premetastatic niche formation and accelerate cancer cell metastasis.^164^ Antiangiogenic agents (e.g., anti‐VEGF antibody, bevacizumab) contribute to normalization of angiogenesis, and their combination with immunotherapy improves the effectiveness of immunotherapy.^165^

VEGF is a key mediator of tumor angiogenesis, which promotes the activation of endothelial cells by binding to receptors on the surface of endothelial cells.^166^ Exosomes secreted by tumor cells promote the secretion of VEGF by human umbilical vein endothelial cells (HUVEC) through various mechanisms. For example, exo‐circ‐0044366 (termed circ29), as a sponge of miR‐29a, inhibits the binding of miR‐29a to VEGF, and promotes the secretion of VEGF.^167^ CircSHKBP1 promotes the expression of HUR, which is an RNA‐binding protein for stabilizing VEGF mRNA.^168^ Y‐box binding protein‐1 (YB‐1) is an important component of inactive messenger RNA particles, which can be released by GC exosomes. After being taken up by HUVEC, it promotes the secretion of angiogenic factors such as IL‐8, VEGF, and MMP‐9.^169^ Hypoxic CRC cells secrete a large number of exosomes to transmit Wnt4, thereby promoting the nuclear translocation of β‐catenin in endothelial cells and activating the canonical Wnt/β‐catenin pathway in cancer to support tumor angiogenesis.^170^ Exo‐miR‐21‐5p promotes nuclear translocation of β‐catenin.^171^

Angiogenesis provides more nutrients for tumor cells, which is obviously beneficial to tumor growth. In addition, blood vessels provide channels for cancer cell metastasis. Based on the importance of exosomes in angiogenesis, the development of drugs that inhibit tumor‐derived exosomes helps to synergize antiangiogenic drugs to inhibit tumor progression.

### 2.10 Potential connections between exosomes and neurons

The role of neurons has often been overlooked in the past when investigating the effects of TME on tumor development. Since it is reported that cancer cells in gliomas connect to neuronal synapses and promote neuronal excitability to modulates active glioma growth, scientists have begun to focus on the roles of neurons in tumor progression.^172^ Cancer cells recruit peripheral nerves to the TME^173^ and inhibit miR‐34a expression to differentiate sensory neurons into adrenergic neurons, thereby supporting tumor progression.^174^ The activation of PI3K‐mTOR pathways is associated with neuronal regulation of tumor cell proliferation.^175^ In the recent years, it has been reported that the mutations in NF1 (multiple neuroangiomatosis‐1) gene modulate the function of hyperpolarization‐activated cyclic nucleotide‐gated channels, thereby causing neurons in tumors to exhibit hyperexcitability. After inhibition with the sodium channel blocker tetrodotoxin or the antiepileptic drug lamotrigine, collagen 1a2 production was reduced in NF1 mutant mice and neurofibroma growth was inhibited.^176^ The number of neurons in TME is closely related to the growth and spread of tumor tissue, which is not only limited to tumors in the brain but also to other types of tumors.^177^

Exosomes play important roles in communication between neurons and other cells. Neuron‐derived exosomes protect the spinal cord from traumatic injury by inhibiting the activation of neurotoxic microglia and astrocytes^178^ and promote M1 polarization of microglia by transferring lncRNA Gas5 to mediate sevoflurane‐induced neurotoxicity in neonatal mice.^179^ MSCs‐derived exosomes reduce the cognitive impairment of progressive Parkinson's disease by altering neuronal cholesterol metabolism through the Wnt5a–LRP1 axis.^180^ The exosome HepaCAM secreted by astrocytes mediates a signaling pathway that stimulates axonal growth in cortical pyramidal neurons.^181^ Based on the regulation of exosomes on neuronal metabolism and growth, it is reasonable that tumor‐derived exosomes also promote their own growth by regulating neurons. Recent studies have preliminarily confirmed this conjecture. Exosomes released by glioma selectively enhance glutamatergic signal transduction by increasing the number of excitatory synapses in patients with brain cancer.^182^

Due to the lack of research on tumor nerves, there is insufficient evidence to elucidate the communication effect of exosomes between tumors and neurons. It is important to explore this direction in the future.

## 3 MECHANISMS OF EXOSOME‐MEDIATED TUMOR IMMUNOTHERAPY

**FIGURE 3:**
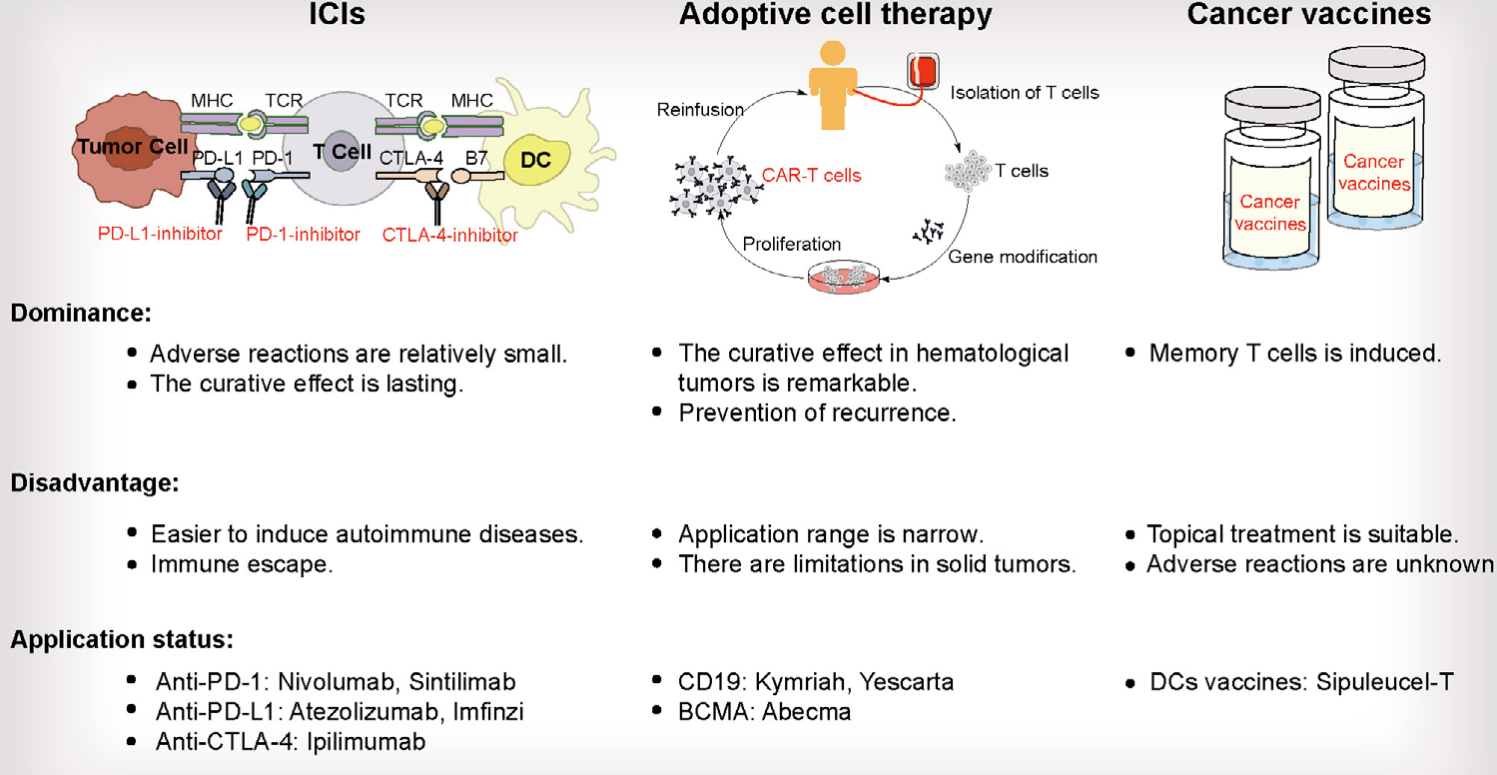
Comparison of multiple immunotherapies. Immunotherapies commonly include ICIs, cancer vaccines and adoptive cell therapy. Different methods have different advantages and disadvantages. Moreover, they have been applied to the clinical treatment of different tumors, and have achieved remarkable results. ICIs, immune checkpoint inhibitors; MHC, major histocompatibility complex; TCR, T cell receptor; PD‐1, programmed cell death protein 1; PD‐L1, programmed death ligand 1; CTLA4, cytotoxic T‐lymphocyte antigen 4; DC, dendritic cell; CAR‐T, chimeric antigen receptor T.

Nowadays, several methods of immunotherapy, including ICIs, cancer vaccines, and adoptive cell therapy, have been utilized in clinical practice and achieved remarkable results (Figure 3). However, some patients showed lower efficacy. Regulation of the TME by exosomes is an important reason why tumor cells escape immunotherapy. Analysis of the relevant mechanisms is essential for exploring breakthrough targets and improving efficacy. In addition, the development of exosomes as drug carriers for synergistic immunotherapy is also worthy of our attention (Table 2).

**TABLE 2:** Preclinical study on the application of exosomes in cancer immunotherapy.

Engineering exosomes	Drug	Function	References
M1 macrophage‐tumor chimeric exosomes	–	Activate T lymphocytes	^199^
CAR‐T exosomes	–	Produce high levels of cytotoxic molecules and inhibit tumor growth	^200^
Engineering MSCs exosomes	miR‐34c‐5p	Inhibit the development of acute myeloid leukemia	^191^
mDexos	BRAF siRNA	Effective treatment of malignant melanoma	^201^
iRGD‐modified exosomes	CPT1A siRNA	Inhibit oxaliplatin resistance	^193^
hGLV	Photothermal agent	Improve macrophage‐mediated tumor cell phagocytosis by blocking CD47 signaling	^202^

Abbreviations: CAR‐T, chimeric antigen receptor T cell; hGLV, hybrid therapeutic nanovesicles by fusing gene‐engineering exosomes with drug‐loaded thermosensitive liposomes.; mDexos, mature dendritic cell exosomes; MSCs, mesenchymal stem cells.

### 3.1 Exosomes and antigen presentation

The significant efficacy of ICIs in the application of solid tumors indicates that immunotherapy has broad development prospects.^183^ However, about 46.4% of cancer patients who respond to ICIs develop acquired resistance during treatment.^184^ The immunosuppressive state of TME is an important reason for the resistance of immunotherapy, such as the decrease of CD8^+^ T cells, as well as the increase of MDSCs and M2 TAMs.^185^ As a communication media, exosomes affect the efficacy of immunotherapy by regulating TME. Exosomes released by immune‐promoting cells can enhance the effect of immunotherapy, while the exosomes of immunosuppressive cells or tumor cells can promote the immune escape of cancer cells.^186^ The exploration of the exosome‐related mechanism in TME is helpful to develop new targets for immunotherapy. Previously, we have discussed the mechanism by which exosomes mediate TME immunosuppression by presenting antigens to regulate immunotherapy. Tumor cells activate the activity of M2‐type TAMs, N1‐type TANs, Treg, MDSCs, and CAFs by releasing exosomes. In return, these cells secrete exosomes containing protumor signaling molecules to support tumor cells. CD8^+^ T cells, B cells, and NK cells are the main force in the antitumor effect, but tumor cells use exosomes to inhibit their antineoplastic activity. Most studies suggest that tumor‐released exosomes promote PD‐L1 expression and TME immunosuppression. Interestingly, in a lung cancer study, tumor‐associated exosomes not only downregulated the expression of PD‐L1 on DCs but also enhanced DCs‐mediated immune response.^187^ At present, the results of this study need to be confirmed by a large number of subsequent studies. The imbalance of tumor‐promoting exosomes and tumor‐inhibiting exosomes in TME promotes tumor cells to evade immunotherapy.^188^ Therefore, targeted inhibition of exosomes released by tumor cells might be an effective method to reactivate the antitumor immunity.

### 3.2 Exosomes as therapeutic agents

The transformation from immunosuppressive “cold tumor” into immune‐promoting “hot tumor” is meaningful to improve the persistence of immunotherapy. Exosomes are a good entry point. They can play a role by utilizing their natural advantages and can also be constructed as engineering exosomes to improve efficacy. Previously, DC‐exosome has been described as a cancer vaccine to exert antitumor effects, which is the most potential natural exosome to improve the efficacy of immunotherapy. Currently, the value of DC‐exosome has been demonstrated in clinical studies. Exosomes derived from NK cells and CD8^+^ T cells contain tumor suppressor factors. For instance, NK‐exo, which relies on miR‐186 expression, is cytotoxic to MYCN‐amplified neuroblastoma cell lines.^189^ Additionally, CD8^+^ T cell‐derived exosomes limit estrogen‐driven endometrial cancer development through the ERβ/miR‐765/PLP2/Notch axis.^190^ This suggests that in vitro derived natural exosomes have potential as therapeutic options for immunotherapy based on NK cells, DCs, and CD8^+^ cells.

**FIGURE 4:**
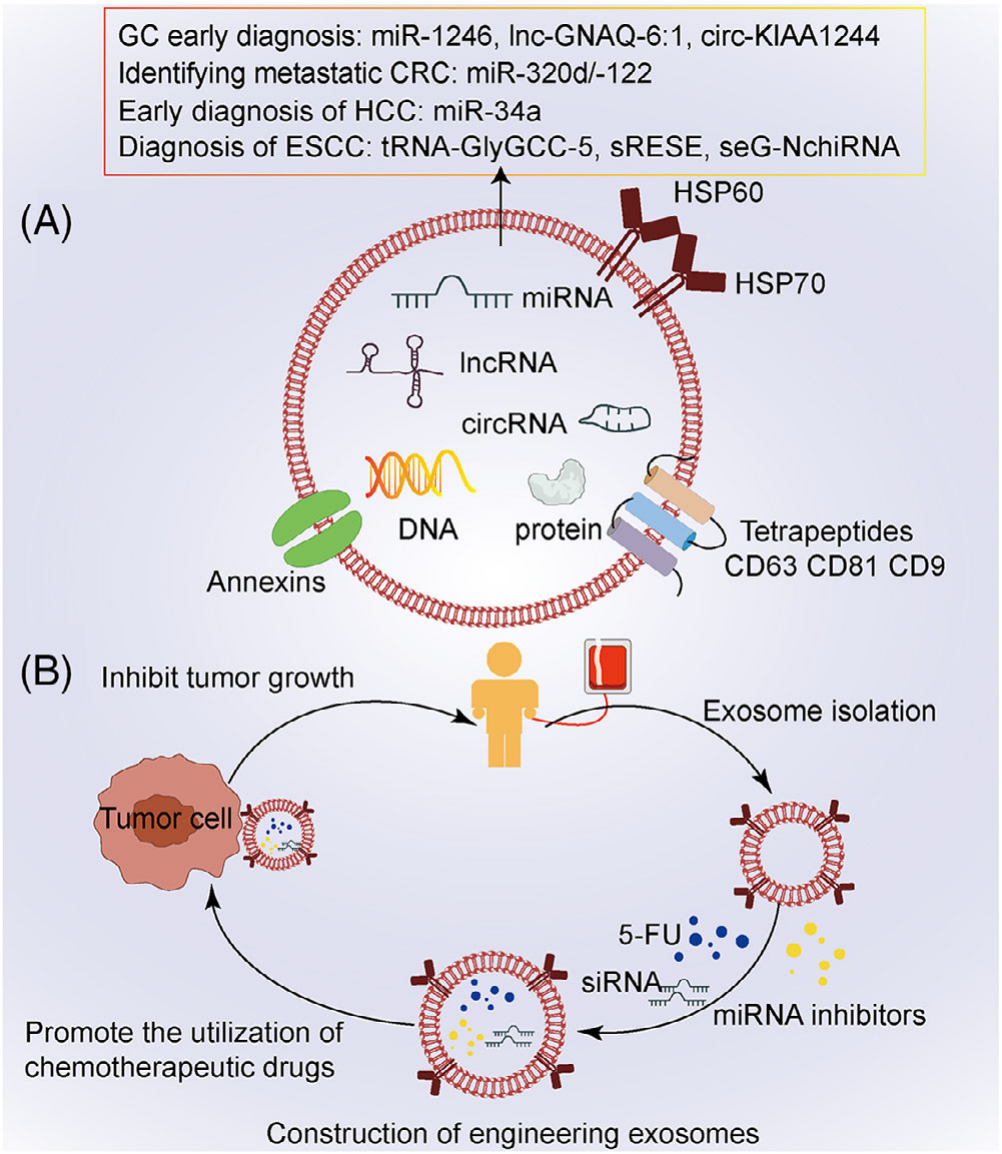
The diagram of exosome and engineering exosome. (A) Composition and structure of exosome. The components of exosome mainly include proteins, nucleic acids, and lipids. Lipid is an important component of exosome membrane structure. The proteins on the surface of exosomes include: annexin, heat shock proteins (HSP60 and HSP70) and tetrapeptides (CD63, CD81, and CD9). Nucleic acids include DNA and RNA. (B) Construction of engineering exosomes. Exosomes are obtained from peripheral blood and loaded with chemotherapeutic drugs, siRNA or miRNA inhibitors to construct engineering exosomes to promote the use of drugs. GC, gastric cancer; CRC, colorectal cancer; HCC, hepatocellular carcinoma; ESCC, esophageal squamous cell carcinoma; HSP60, heat shock protein 60; HSP70, heat shock protein 60; 5‐FU, 5‐fluorouracil; siRNA, small interfering RNA.

Engineering exosomes are designed as the drug delivery system based on the targeting and transportability of exosomes (Figure 4). In recent years, with the continuous refinement and diversification of various technologies of engineering exosomes, more and more researchers have begun to pay attention to their role in tumor treatment. For example, engineering exosomes loaded with miR‐34c‐5p promote the elimination of leukemia stem cells and inhibit the development of acute myeloid leukemia.^191^ This is a potential stem cell replacement therapy. The delivery system based on exosome effectively promote the uptake of drugs by tumor cells and inhibited resistance.^192^ The iRGD‐modified engineering exosomes can effectively target CRC cells and deliver CPT1A siRNA, which inhibits oxaliplatin resistance by regulating fatty acid oxidation.^193^ Sorafenib is the first‐line treatment for advanced HCC, and its resistance is mainly related to GPX4 and DHODH. The engineering exosomes targeting these two genes can be used to overcome the sorafenib resistance.^194^ A variety of miRNAs play a catalytic role in tumor progression, so exosomes loaded with miRNA inhibitors also achieve the effect of treating tumors, such as miR‐21.^192^ Exosomes secreted by tumor cells and stromal cells in TME play an important role in the malignant phenotype of tumor cells. Inhibition of exosome secretion can effectively inhibit tumor progression. GW4869 and GW7721 are inhibitors of exosomes, which not only reduce the migration caused by hypoxic exosomes and the invasion of normoxic CRC cells,^195^ but also reduce the induction of M2 macrophages.^122^ In addition, some researchers began to use the constructed engineering exosomes to program the TME to achieve the effect of treating cancer. The mechanisms involved in this process include the promotion of M1 polarization^196^ and the induction of ferroptosis in tumor cells.^197^

Recent studies have combined engineering exosomes with immunotherapy to create a drug delivery system based on CAR‐T cells or CAR‐NK cell‐derived exosomes for the treatment of cancer.^45^, ^74^ This research shows great promise. However, exosomes as a drug‐loading system for the treatment of tumors have problems such as low efficiency and large drug loss. The reprogramming of tumor exosome vector by low pH not only retains the specificity of its related cells but also improves the uptake efficiency.^198^ At present, researchers are still committed to finding a solution to improve the efficiency of exosome‐loaded systems.

## 4 CLINICAL APPLICATIONS AND CHALLENGES OF EXOSOME‐BASED CANCER IMMUNOTHERAPY

With the continuous growth of exosome‐related research, researchers' attention has shifted from the pathogenic mechanism of exosomes to the study of their diagnostic and therapeutic value. Relevant clinical studies are being conducted progressively.

### 4.1 The clinical application and challenges of exosome‐based tumor diagnosis

Due to the lipid bilayer, the biomolecules contained in exosomes can stably exist in body fluids and are easily obtained. Exosomes secreted by tumor cells are specific. The natural advantages of exosomes make them show great potential in the treatment of tumors. Compared with other biomarkers, exosome molecules may more accurately reflect the disease status, evaluate the drug response, and distinguish metastatic cancer.^203^ We summarize the latest research on the diagnostic value of exosome biomolecules in tumors (Table 3). The single marker usually has the diagnostic efficacy with low sensitivity or specificity. The construction of molecular marker models may break this deadlock. The model (including let‐7i‐5p, miR‐1307‐3p, LZIC, SRSF6, lncFTH1‐211, and lncPTMA‐209) have 100% sensitivity and specificity for determining the efficacy of fluorouracil‐oxalate combined neoadjuvant chemotherapy (NACT) in advanced GC.^204^ In addition, this situation may also be caused by the low sensitivity of the nucleic acid detection methods. The traditional nucleic acid detection methods rely on real‐time quantitative polymerase chain reaction. This method has certain limitations. It cannot directly detect nucleic acid level and requires RNA purification first. The platform was constructed by transfecting clustered regularly interspaced short palindromic repeats (CRISPR)‐associated proteins (CRISPR/Cas) into exosomes, which can directly detect plasma exosomal miRNA.^205^ The platform achieves a linear range of four orders of magnitude for the detection of miR‐21, which effectively improves the sensitivity of exosomal miRNA detection.^205^ Recently, in order to aid in the early identification of pancreatic cancer, researchers have developed a dual‐specific biomarker antigen corecognition and capture strategy to detect target tumor exosomes by grafting two corresponding capture antibodies on magnetic nanoparticles and gold nanoparticles. This method's excellent specificity and ultra‐high sensitivity allow it to detect the pancreatic cancer exosome‐specific protein glypican‐1 as low as 78 pg/mL.^206^

**TABLE 3:** Prospective diagnostic markers and models in exosome‐related clinical trial.

Exosome‐derived molecules (or model)	Sample	Function	Case/Control	AUC (95% CI)	Sensitivity	Specificity	Reference
A 6‐exosome‐RNA biomarker model	Plasma	To identify the efficacy of NACT in advanced GC	29/34	1.00 (NR)	1.00	1.00	^204^
miR‐320d	Serum	To distinguish between metastatic and non‐metastatic CRC patients	34/108	0.63 (NR)	NR	NR	^212^
miR‐1246	Serum	GC early diagnosis	85/50	0.91 (0.86–0.96)	0.82	0.86	^213^
miR‐363‐5p	Plasma	To predict the prognosis and lymph node metastasis of BC	10/10	0.96 (NR)	NR	NR	^214^
miR‐1290	Serum	Diagnosis of lung adenocarcinoma	70/40	0.94 (0.90–0.99)	0.80	0.97	^215^
hsa‐miR‐21‐5p	Plasma	Diagnosis of BC	30/30	0.96 (0.92–1.00)	0.87	0.93	^216^
CEA+lncRNA GAS5	Serum	Early diagnosis of NSCLC	64/40	0.93 (0.88–0.98)	0.89	0.90	^217^
miRNA‐1307‐5p	Salivary	Poor prognostic markers of oral cancer	12/5	0.99 (NR)	1.00	1.00	^218^
miR‐4732‐5p	Plasma	Diagnostic markers of epithelial OC	34/21	0.89 (NR)	0.86	0.82	^219^
miR‐34a	Serum	Early diagnosis of HCC	60/60	0.66 (0.57–0.75)	0.78	0.52	^220^
circ‐PNN	Serum	Diagnosis of CRC	88/88	0.86 (0.79–0.90)	0.90	0.74	^221^
hsa‐circ‐0004771	Serum	To discriminate stage I/II CRC patients from healthy controls	110/35	0.86 (0.79–0.93)	0.81	0.80	^222^
bi‐sesncRNA signature (tRNA‐GlyGCC‐5, sRESE)	Saliva	Diagnosis of ESCC	200/120	0.93 (NR)	0.91	0.94	^223^
seG‐NchiRNA	Saliva	To detect early and advanced‐stage ESCC	61/159	0.91 (NR)	0.89	0.89	^224^
MT1‐MMP	Serum	Diagnosis of GC	216/16	0.79 (0.71–0.85)	0.64	0.87	^225^
ITGA6	Plasma	The early screening of CRC	14/28	0.84 (0.68–0.99)	NR	NR	^226^
ITGB3	Plasma	The early screening of CRC	14/28	0.78 (0.58–0.99)	NR	NR	^226^
Cx43	Plasma	Diagnosis of melanoma	112/50	0.78 (0.70–0.86)	0.82	0.78	^227^

Abbreviations: BC, breast carcinoma; CEA, carcinoembryonic antigen; CRC, colorectal cancer; Cx43, connexin 43.; ESCC, esophageal squamous cell carcinoma; GC, gastric cancer; G‐NchiRNA, salivary exosomal GOLM1‐NAA35 chimeric RNA; HCC, hepatocellular carcinoma; NACT, neoadjuvant chemotherapy; NSCLC, non‐small cell lung cancer; OC, ovarian cancer.

The diagnostic potential of exosomes is widely recognized, but their usefulness is limited by the shortcomings of single detection methods and low sensitivity. As a result, exosomes are unable to fully realize their potential. Currently, researchers are developing new detection methods and have achieved some promising results. The main principle of these methods is to improve sensitivity by expanding the linear range of detection. These research findings further promote the clinical application of exosomes.

### 4.2 Clinical applications and challenges of exosome‐based cancer immunotherapy

Preclinical studies have demonstrated that both natural and engineering exosomes can enhance the effectiveness of immunotherapy. This is promising news for patients who have no response to immunotherapy. At present, clinical studies based on exosome immunotherapy mainly focus on cancer vaccines constructed by DC‐exosome, and more clinical studies are in the registration and trial stage. As early as 2005, researchers used DC‐exosome to load MAGE tumor antigen to treat NSCLC. This clinical study confirmed that DC‐exosome vaccine is feasible and DC‐exosome treatment is well tolerated.^207^ Subsequent phase II experiments confirmed that DC‐exosomes act by enhancing the antitumor activity of NK cells.^208^ In addition, the safety and feasibility of the vaccine have also been confirmed in clinical studies of metastatic melanoma,^209^ CRC,^210^ and EC.^211^ Recently, other clinical studies of exosome‐based immunotherapy are in full swing. For example, researchers have used exosomes to carry therapeutic targets (such as CD20, PDL‐1) as “bait receptors” for immunotherapy to clarify the role of exosomes in the immunotherapy of non‐Hodgkin B‐cell lymphomas (NCT03985696).

Clinical studies have confirmed the safety and reliability of exosome‐based immunotherapy. However, the available clinical data are still insufficient. Further research is needed before exosome‐related drugs can be used to treat cancer patients. This direction is necessary for researchers to continue exploring and devoting themselves to research. Exosome‐based cancer immunotherapy has unlimited potential, which is currently the most promising clinical treatment method, but there are some obstacles. The industrialization of DC‐exosome cancer vaccines or engineering exosomes requires large‐scale exosomes. Current exosome isolation techniques include ultracentrifugation, ultrafiltration, polymer precipitation, immunoaffinity chromatography, size‐exclusion chromatography, and microfluidic‐based methods. These technologies are not enough to extract large amounts of exosomes or have low extraction efficiency. Furthermore, the heterogeneity of exosomes can impact the efficiency of extraction using these technologies and the purity of the resulting exosomes. Therefore, it is necessary to continue developing new extraction methods or optimizing the existing ones to achieve the industrialization of exosomes. This will enable the use of exosome‐based therapeutic drugs in clinical practice.

## 5 CONCLUSION AND PROSPECTS

**FIGURE 5:**
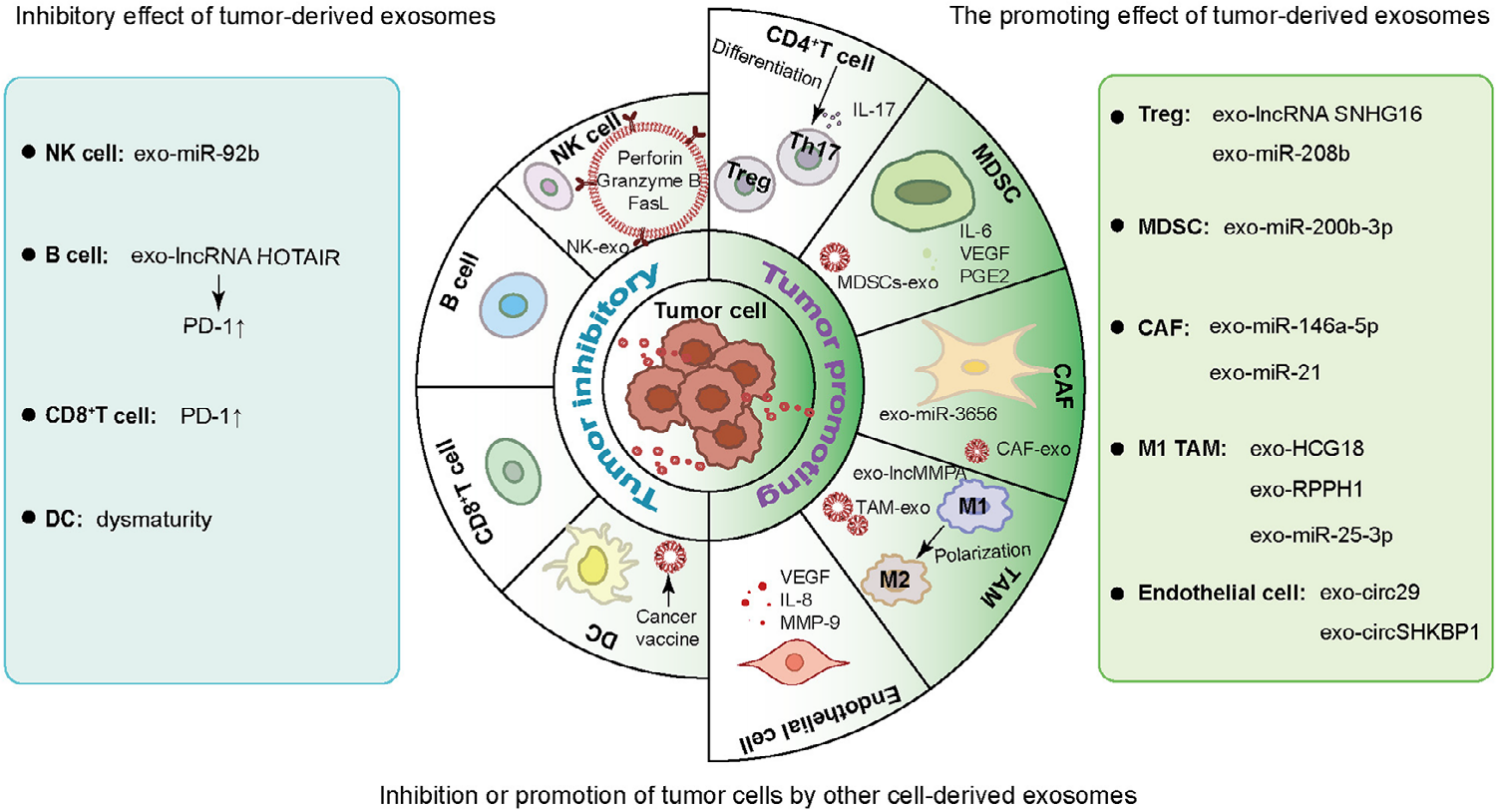
The role of exosomes in the communications between various components of TME. Tumor cells use exosome signals to inhibit the activity of cytotoxic immune cells and promote the proliferation and differentiation of cells with tumor‐promoting activity, thereby inducing immune escape of tumor cells. Tumor promoting immune cells (M2 TAMs, MDSCs) and CAFs further enhance the immune escape of cancer cells by secreting exosomes. In contrast, the tumor‐killing cells employ exosomes to inhibit tumor cells. NK, natural killer; DC, dendritic cell; Treg, regulatory T cell; Th17, T helper type 17; MDSC, myeloid‐derived suppressor cell; CAF, cancer‐associated fibroblast; TAM, tumor‐associated macrophage; exo, exosome; PD‐1, programmed cell death protein 1; VEGF, vascular endothelial growth factor; FasL, Fas ligand.

Immunotherapy is a promising method for treating cancer, but in some cases, it does not produce a positive therapeutic response. One of the main obstacles is that infiltrating cells in TME protect cancer cells from treatment. The mechanism of the communications between cancer cells and infiltrating cells in TME is very complex. Exosome is an important connection hub for communication between various components of TME (Figure 5). Due to the significance of exosomes, researchers are currently developing immunotherapy methods based on exosomes and have made notable progress. Current studies reveal that exosomes from DCs or NK cells have the cytotoxicity of parental cells and can be developed as a cancer vaccine to alleviate tumor progression.^81^ The vaccine has demonstrated some efficacy in clinical studies. However, to industrialize it, it is necessary to continuously optimize the extraction method of exosomes. In addition, the natural advantages of exosomes make them be widely studied as drug carriers.^228^ Using the antigen presentation function and protective effect, DC‐exosome can be used as the targeted delivery of drugs in tumor treatment to reduce drug loss and promote the effect of immunotherapy.^229^ Because of the lack of sufficient evidence, it is unknown whether the exosomes from other antitumor cells (such as CD8^+^ T cells, CD4^+^ T and B cells) in tumors can be used as cancer vaccines or drug delivery system. The latest research suggests that the integration of engineering exosomes with other therapeutic methods is a promising research direction in the future.

Moreover, exosomes from tumor cells might be developed as diagnostic or prognostic markers. Based on current studies, some exosome molecules secreted by tumor cells are more specific and accurate for monitoring the disease status compared with other biomarkers.^230^ It is also reported that exosomes can be used to evaluate drug response and distinguish metastatic cancer for precise cancer treatment.^203^ However, the clinical practices of exosomes in diagnosis and treatment need to be further determined. Some challenges need to be solved in exosome research. The methods for isolating and identifying exosomes are complicated and inconsistent. The convenient operation process and unified standard are critical for clinical practice. Moreover, the heterogeneity of exosomes includes size heterogeneity, content heterogeneity, source heterogeneity, and functional heterogeneity. It is important to identify the specific exosome in tumor progression. The mechanism of production, maturation, secretion, and activity of exosome are complicated and under determination. In the future, it is important to address these issues in order to more deeply understand the biological roles of exosomes in tumor progression and increase the possibility of exosome‐related clinical use. The research prospects of exosomes are still broad and its potential will be unlimited.

## AUTHOR CONTRIBUTIONS

*Writing—original draft preparation*: Yu Wu, Wenyan Han, and Hairong Dong. *Writing—review and editing*: Xiaofeng Liu and Xiulan Su. All authors have read and agreed to the published version of the manuscript.

## CONFLICT OF INTEREST STATEMENT

The authors declare no conflict of interest.

## ETHICS STATEMENT

Not applicable.

## Data Availability

Not applicable.
